# A Breath of Life Beyond Bronchodilation: Early Data on Hemodynamic and Functional Outcomes With Ensifentrine Nebulization in Severe COPD-associated Pulmonary Hypertension

**DOI:** 10.2174/0118743064456019260621185404

**Published:** 2026-07-01

**Authors:** Ramakanth Pata, Joanna Kristeva, Mallory Illies, Justin Stocks

**Affiliations:** 1 Pulmonary and Critical Care Medicine CentraCare Health System, MN, USA

**Keywords:** COPD, Pulmonary hypertension, Ensifentrine, Phosphodiesterase inhibitor, Retrospective study, 6-minute walk distance, Right heart catheterization

## Abstract

**Introduction:**

Pulmonary hypertension (PH) is a frequent and serious complication of advanced chronic obstructive pulmonary disease (COPD) and is associated with a poor prognosis and limited treatment options. Current management focuses on optimizing COPD care, while the role of pulmonary vasodilators remains controversial. Ensifentrine, a dual phosphodiesterase (PDE) 3/4 inhibitor with bronchodilatory and anti-inflammatory properties, may offer a therapeutic benefit in COPD-associated PH.

This study aims to retrospectively evaluate the effects of six months of nebulized ensifentrine on pulmonary hemodynamics and functional outcomes in patients with severe COPD and associated pre-capillary PH.

**Methods:**

We conducted a single-center, retrospective chart review of patients with GOLD stage III and IV COPD and confirmed pre-capillary PH (mean pulmonary artery pressure [mPAP] ≥20 mmHg, pulmonary vascular resistance [PVR] >2 Wood units, pulmonary capillary wedge pressure ≤15 mmHg). Five patients treated with nebulized ensifentrine (3 mg twice daily) between September 2024 and May 2025 were included. Hemodynamics *via* right heart catheterization (RHC) and 6-minute walk distance (6MWD) were measured at baseline and at 6 months.

**Results:**

The cohort (mean age 76 years, 60% male, mean FEV_1_ 33.4% predicted) demonstrated severe emphysema and PH (baseline mPAP 53.2 mmHg, PVR 9.5 WU, cardiac index 2.5 L/min/m^2^). After 6 months, mean changes were as follows: • mPAP: decreased by 1.6 mmHg • Cardiac index: increased by 0.1 L/min/m^2^ • PVR: decreased by 1.0 WU • 6MWD: improved by 31 m • mMRC dyspnea score improved from 3.6 to 3.2 • CAT score from 23 to 21.6 • DLCO increased modestly (29%→31.2% predicted). • No adverse events, exacerbations, or hospitalizations were observed during this period. • The combination of ensifentrine with roflumilast was well tolerated.

**Discussion:**

Pulmonary hypertension in COPD is associated with worse outcomes, yet treatment options remain limited and uncertain. In this small retrospective case series of five patients with very severe COPD and confirmed pre-capillary PH, the addition of ensifentrine to optimized therapy was associated with modest but potentially meaningful improvements in symptoms, exercise capacity (6MWD), and select hemodynamic parameters, without worsening oxygenation. Its dual PDE3/PDE4 inhibition and inhaled delivery provide a plausible mechanism for bronchodilation, anti-inflammatory effects, and selective pulmonary vasodilation while minimizing ventilation–perfusion mismatch. However, the findings are highly preliminary given the very small sample size, lack of a control group, retrospective design, and significant selection bias, limiting generalizability and causal inference. Overall, the results are hypothesis-generating and suggest that ensifentrine may have therapeutic potential in COPD-associated PH, but larger, prospective, randomized trials are needed to confirm efficacy, safety, and its role in treatment algorithms.

**Conclusion:**

In this retrospective study, ensifentrine was safe and was associated with modest improvements in pulmonary hemodynamics and functional status in patients with severe COPD with pre-capillary PH. These preliminary findings warrant confirmation in larger, prospective, controlled trials.

## 1 INTRODUCTION

Chronic obstructive pulmonary disease (COPD) is a leading cause of death worldwide, ranking fourth globally and responsible for approximately 3.5 million deaths in 2021 (≈5% of all deaths). Nearly 90% of COPD-related deaths among individuals under 70 years of age occur in low- and middle-income countries (LMICs). COPD is also a major contributor to poor health, ranking eighth in global disability-adjusted life years. Tobacco smoking accounts for over 70% of cases in high-income countries, but only 30–40% in LMICs, where household air pollution is an important additional risk factor [1]. In the United States, COPD is among the top ten causes of death, affecting nearly 16 million adults, many of whom remain undiagnosed [2]. Modeling studies predict that the global number of COPD cases among individuals aged ≥25 years will increase by 23% from 2020 to 2050, approaching 600 million cases. The projected burden is expected to rise most sharply among women, with a 47.1% increase compared with 9.4% for men, and to be disproportionately higher in LMICs, where the number of cases is expected to more than double that in high-income regions by 2050 [3]. These data highlight the growing global health challenge posed by COPD, particularly for women and populations in resource-limited settings.

Pulmonary hypertension (PH) is a well-recognized complication of advanced chronic obstructive pulmonary disease (COPD), classified under WHO Group 3 PH. The prevalence of pulmonary hypertension in patients with COPD is estimated to range from 13% to 64%, with a pooled prevalence of around 39% reported in a meta-analysis [2, 4]. It arises from chronic hypoxic vasoconstriction and pulmonary vascular remodeling, and contributes to dyspnea, reduced exercise tolerance, right ventricular dysfunction, and excess mortality. Despite its clinical significance, therapeutic options remain limited, with most guidelines recommending optimization of COPD management and long-term oxygen therapy as the mainstay of treatment [5].

Systemic pulmonary vasodilators have shown mixed results in COPD-associated PH. While phosphodiesterase-5 inhibitors and endothelin receptor antagonists may improve hemodynamics, their clinical benefit is inconsistent, and concerns remain regarding ventilation–perfusion mismatch and worsening hypoxemia. Consequently, such agents are typically reserved for clinical trials or highly selected cases in specialized centers [6].

Ensifentrine is a novel inhaled dual PDE3/4 inhibitor with bronchodilatory and anti-inflammatory effects. Recent phase III trials (ENHANCE-1 and ENHANCE-2) demonstrated improvements in lung function, quality of life, and symptom burden in moderate-to-severe COPD. However, its effects on pulmonary hemodynamics in COPD patients with confirmed PH have not been previously studied [7].

This retrospective study describes the hemodynamic and functional outcomes in a small cohort of patients with severe COPD and pre-capillary PH treated with nebulized ensifentrine for six months.

## 2 MATERIALS AND METHODS

### 2.1 Study Design and Setting

This single-center, retrospective observational study included patients treated with ensifentrine between September 2024 and May 2025 at a specialized COPD-PH clinic. Institutional Review Board approval was obtained, and informed consent was secured from all participants before data extraction. The study was conducted in accordance with the Declaration of Helsinki.

### 2.2 Patient Selection

Medical records of 14 patients with end-stage COPD and suspected PH were reviewed. Nine patients were excluded for the following reasons:

- Heart failure with preserved ejection fraction (HFpEF) confirmed on right heart catheterization (RHC) (n = 2)
- Severe exacerbation or hospitalization during follow-up (n = 2)
- New acute coronary syndrome (n = 1) during the 6-month observational period
- Endobronchial valve placement (n = 1)
- Logistic inability to repeat RHC (n = 2)
- Intolerable adverse effects of ensifentrine (nightmares, n = 1)

The final cohort comprised 5 patients meeting the following inclusion criteria:

- Age ≥18 years
- GOLD (Global Initiative of Chronic Obstructive lung diseaseLung Disease) stage III–IV COPD (FEV_1_ <50% predicted)
- Six-minute walk distance (6MWD) <330 m
- Pre-capillary PH confirmed by RHC: mPAP (mean pulmonary artery pressure) ≥20 mmHg, PVR (pulmonary vascular resistance) > 2 WU (Wood units), PCWP (Pulmonary Capillary wedge pressure) ≤15 mmHg
- Stable and maximal guideline-based COPD therapy ≥ 6 weeks before ensifentrine initiation.

All patients were recommended to participate in pulmonary rehabilitation for at least three months before initiation of ensifentrine as part of standard clinical management. The majority of patients (4 of 5) completed pulmonary rehabilitation; however, one patient declined participation but was included in the analysis.

Exclusion criteria included left heart disease, chronic thromboembolic PH, interstitial lung disease, bronchiectasis, active smoking, systemic autoimmune disease, changes in background COPD therapy, and candidacy for lung transplantation. The study flow chart has been given as Fig. (**1**).

### 2.3 Intervention

Patients were maintained on stable, guideline-directed COPD therapy for at least three months before initiation of ensifentrine, and no major therapy changes occurred during the observation period whenever possible. Patients received nebulized ensifentrine (3 mg twice daily) *via* a standard nebulizer device for six months, in addition to maximal guideline-directed COPD therapy. Four patients were receiving roflumilast, and all were counseled regarding potential additive adverse effects.

### 2.4 Data Collection

Baseline demographics, comorbidities, medications, pulmonary function tests (PFTs), echocardiography, right heart catheterization (RHC), six-minute walk distance (6MWD), and patient-reported outcomes (modified Medical Research Council [mMRC] dyspnea scale and COPD Assessment Test [CAT]) were collected. RHC and 6MWD were repeated at six months. Adverse events and exacerbations were also recorded.

### 2.5 Statistical Analysis

Given the small sample size, outcomes were analyzed descriptively (means ± standard deviation where applicable). No hypothesis testing was performed. Individual patient-level data trends were reviewed to assess consistency.

## 3 RESULTS

### 3.1 Baseline Characteristics

The cohort included 5 patients (mean age 76 years, 60% male, BMI 19.6, Charlson Comorbidity Index- 9.6). All were former heavy smokers (mean 47 pack-years) and had severe emphysema on imaging (See Fig. (**2**) for a representative Chest CT of one of the patients). Two out of five reported moderate exacerbations and another two severe exacerbations in the previous year. Clinical and radiologic evaluation suggested a bronchitic phenotype in 3 of 5 patients in addition to severe emphysema. Bronchitic phenotype was defined by the clinical history of persistent cough associated with mucus production for at least 3 months in the previous 2 years. Baseline mMRC was 3.6, CAT 23, mean FEV1 (FEV: Forced Expiratory Volume) 33.4% predicted, and DLCO (Diffusion Limited Carbon Monoxide) 29% predicted. The mean Hb was 14.6 g/dL, and the absolute eosinophil count was 210/mm^3^. As a part of evaluation for worsening exertional dyspnea, all underwent echocardiography, which revealed pulmonary hypertension with a mean ePASP (estimated pulmonary artery systolic pressure) of 62.4 mm Hg and a mean TAPSE (tricuspid annular plane systolic excursion) of 1.42 cm. BNP (B-type natriuretic peptide) averaged 90.4 pg/mL. The mean 6MWD at the time of initial evaluation was 224m. All were deemed not candidates for lung transplantation. Table **1** presents the baseline characteristics.

All participants were receiving guideline-directed therapy, including inhaled LABA-LAMA-ICS combinations (one patient was on LAMA+ LABA only), oral roflumilast (n = 4), chronic azithromycin (n = 4), chronic prednisone (n = 3), and subcutaneous dupilumab (n = 1). Four of five patients had participated in a pulmonary rehabilitation program before enrollment; one patient declined pulmonary rehabilitation. Patients with left heart disease, chronic thromboembolic PH, or systemic inflammatory conditions (including autoimmune conditions) were excluded. Four patients used positive expiratory pressure (PEP) devices and non-invasive ventilation. Oxygen-requiring. All five patients required supplemental oxygen at 1–2 L/min with exertion, and none required oxygen at rest, which was negative for Ischemia. The fifth patient had no significant coronary artery calcification on the CT Chest.

Two patients underwent coronary angiography two years prior, which showed no significant obstructive coronary lesions. Two patients had stress testing that was negative for ischemia. The fifth patient had no significant coronary artery calcification on chest CT.

Patients who had HFpEF were excluded. All patients were initially assessed using the H_2_FPEF score, a validated clinical scoring system incorporating body mass index, use of ≥2 antihypertensive medications, atrial fibrillation, pulmonary artery systolic pressure, age >60 years, and echocardiographic E/e′ ratio. Patients with a low H_2_FPEF score (0–1) were considered unlikely to have HFpEF [8]. In patients with an intermediate probability of HFpEF, invasive hemodynamic assessment was performed using right heart catheterization (RHC) with a standardized fluid challenge.

RHC was used to measure right atrial pressure, pulmonary artery pressures, and pulmonary capillary wedge pressure (PCWP). The fluid challenge consisted of rapid infusion of 500 mL of normal saline over approximately 5–10 minutes, followed by repeat measurement of PCWP. An increase in PCWP to ≥18 mmHg after fluid challenge was considered consistent with occult HFpEF [9, 10]. Patients meeting these hemodynamic criteria were classified as having HFpEF and were excluded from the study.

Right heart catheterization in five patients revealed severe pulmonary hypertension, with a mean pulmonary artery pressure of 53.2 mmHg, a mean pulmonary capillary wedge pressure of 10.2 mmHg, pulmonary vascular resistance of 9.5 Wood units, and a mean cardiac index of 2.5 L/min/m^2^. Measurements were obtained at end-expiration in three patients, while mean cycle values were used in two patients due to marked respiratory pressure variability.

Patients received nebulized ensifentrine (3 mg twice daily *via* a standard nebulizer) for six months, initiated within three months of the initial right heart catheterization. This was undertaken before a planned, individualized trial of sildenafil, as ensifentrine had received FDA approval at that time, and a trial of sildenafil was therefore deferred.

Patients already receiving roflumilast were extensively counseled regarding the potential adverse effects of the combination of roflumilast and ensifentrine as part of a shared decision-making process. Ensifentrine was considered a salvage therapy in patients already on roflumilast before consideration of sildenafil. This involved an extensive risk–benefit discussion, and treatment was initiated only in patients who agreed to close surveillance *via* electronic patient portal messaging and structured questionnaires. Patients were also closely monitored for liver dysfunction, weight loss, and depression.

Repeat right heart catheterization was performed approximately six months after initiation of ensifentrine.

### 3.2 Findings

A clinic follow-up visit was performed six months after initiation of ensifentrine, at the time of repeat right heart catheterization. Patients reported improvement predominantly in dyspnea rather than cough or sputum production, corresponding to a reduction in mMRC score from a mean of 3.6 to 3.2 and CAT score from 23 to 21.6 There were no differences in exacerbation rates compared with the preceding six months.

Pulmonary function testing demonstrated a mean FEV_1_ of 33.2% predicted, FVC of 80.2% predicted, and DLCO of 31.2% predicted. A follow-up 6-minute walk distance (6MWD) showed improvement from a mean of 224 m to 255 m.

Repeat right heart catheterization (performed in all patients; three also underwent simultaneous left heart catheterization due to concern for right ventricular dysfunction, which revealed non-significant coronary artery disease) demonstrated a reduction in mean pulmonary artery pressure from 53.2 to 51.6 mmHg, a similar mean PCWP of 9.8 mmHg, and a decrease in mean PVR from 9.5 to 8.5 Wood units. Cardiac index increased from 2.5 to 2.6 L/min/m^2^. No adverse events or hospitalizations were documented.

At the six-month follow-up after repeat hemodynamic assessment, all five patients were started on a trial of sildenafil therapy. Two patients discontinued sildenafil after one month due to dizziness and perceived worsening oxygenation. The remaining three patients tolerated the medication without significant symptomatic change at three-month follow-up.

### 3.3 Safety and Tolerability

No adverse events, including weight loss >5%, abnormal liver function tests, or new-onset or worsening depression, were observed. There was no change in the frequency of COPD exacerbations or hospitalizations compared with the same 6-month period in the previous year. The combination of roflumilast and ensifentrine was well tolerated by all patients.

Of note, one patient (not included in this study) discontinued ensifentrine nebulization within one week due to adverse effects, including nightmares and vivid dreams involving snakes. This patient was already receiving roflumilast.

### 3.4 Outcomes After 6 Months of Ensifentrine

- Hemodynamics: mPAP decreased from 53.2 to 51.6 mmHg; PVR decreased from 9.5 to 8.5 Wood units; cardiac index increased from 2.5 to 2.6 L/min/m^2^; PCWP remained stable (10.2 to 9.8 mmHg).
- Functional capacity: 6-minute walk distance (6MWD) improved from 224 to 255 m (+31 m).
- Patient-reported outcomes: mMRC decreased from 3.6 to 3.2; CAT score from 23 to 21.6.
- Pulmonary function tests: FEV_1_ remained stable (33.4% to 33.2% predicted), while DLCO showed a modest improvement (29% to 31.2% predicted).
- Safety: No weight loss >5%, liver dysfunction, depression, or increased exacerbations were observed. No hospitalizations occurred. The combination with roflumilast was well tolerated.

Table **2** presents hemodynamic and functional outcomes at baseline and six months after ensifentrine nebulization.

## 4 DISCUSSION

Pulmonary hypertension (PH) in chronic obstructive pulmonary disease (COPD) is independently associated with reduced survival, higher rates of exacerbations, and impaired quality of life [11]. Even in the setting of maximized inhaled therapy, many patients continue to experience progressive dyspnea and exercise intolerance, driven by pulmonary vascular remodeling and increased right ventricular (RV) afterload, which may ultimately lead to chronic RV dysfunction [12].

The management of COPD-associated PH (COPD-PH) remains an area of considerable uncertainty, as no therapies are currently approved specifically for this indication. Current guidelines emphasize optimization of underlying COPD therapy, initiation of long-term oxygen therapy in selected patients, and reserve the use of pulmonary vasodilators for carefully selected cases within specialized centers [5].

Patients with Group 3 PH, especially those with severe airflow obstruction and gas exchange impairment, have historically been excluded from most pulmonary arterial hypertension (PAH) trials. As a result, clinicians are left with limited guidance and few therapeutic options. Evidence for pulmonary vasodilator therapy in COPD-associated PH remains conflicting [6]. Our preliminary observations suggest a potential signal for the utility of ensifentrine in this population; however, these exploratory findings should be interpreted cautiously and warrant further investigation in larger prospective studies.

In this retrospective cohort, all five patients had very severe COPD (mean FEV_1_ 33.4% predicted). Four of five were receiving domiciliary noninvasive ventilation, and all had severe pre-capillary PH with a mean pulmonary vascular resistance (PVR) of 9.5 Wood units. Echocardiographic and hemodynamic data demonstrated evidence of right ventricular (RV) dysfunction, with a mean tricuspid annular plane systolic excursion (TAPSE) of 1.42 cm and a cardiac index of 2.5 L/min/m^2^, in the absence of significant coronary artery disease or HFpEF. These findings highlight the adverse right ventricular remodeling that can occur in longstanding COPD-associated PH, resembling a phenotype of “COPD-associated RV cardiomyopathy.” Functional impairment was profound, as demonstrated by a mean six-minute walk distance (6MWD) of only 224 m despite optimized medical therapy and a severe emphysematous phenotype.

Traditional pulmonary vasodilators, such as endothelin receptor antagonists and phosphodiesterase-5 inhibitors (PDE5i, *e.g.*, sildenafil), are generally avoided in COPD due to concerns regarding ventilation-perfusion (V/Q) mismatch and hypoxemia, although they may be considered on a case-by-case basis in expert centers or within clinical trials [6]. Previous studies of sildenafil in COPD-associated PH have yielded conflicting results, with consistent improvements in pulmonary hemodynamics but mixed effects on exercise capacity and oxygenation. A central concern with systemic vasodilators is their potential to exacerbate V/Q mismatch through nonselective vasodilation of poorly ventilated lung regions [4]. By contrast, ensifentrine’s inhaled delivery may mitigate this risk by preferentially targeting better-ventilated areas of the lung.

Preclinical and phase II studies support the mechanistic rationale for PDE3 and PDE4 inhibition, demonstrating dose-dependent relaxation of airway smooth muscle and anti-inflammatory effects [13]. PDE3 regulates cAMP and cGMP signaling in airway smooth muscle, thereby influencing bronchial tone [7]. PDE3 inhibition is also known to increase myocardial contractility *via* reduced cAMP degradation and to promote pulmonary vasodilation *via* effects on cAMP and cGMP signaling (*e.g.*, milrinone) [14].

In our cohort, the addition of nebulized ensifentrine to standard therapy was associated with subjective improve-ment in dyspnea, as reflected in reduced modified Medical Research Council (mMRC) and COPD Assessment Test (CAT) scores, although cough and sputum production remained largely unchanged. All patients had a predom-inant severe emphysema phenotype with dyspnea as the leading symptom; none had coexistent bronchiectasis, and three demonstrated chronic bronchitic features.

Hemodynamic changes were modest, with a mean reduction in PVR of 1 Wood unit and an increase in cardiac index of 0.1 L/min/m^2^. These findings may reflect either biological effect or measurement variability; however, the consistency of improvement across multiple domains, including 6-minute walk distance (6MWD) and diffusing capacity for carbon monoxide (DLCO), supports biological plausibility. On average, 6MWD improved by 31 m. While modest, this improvement may be clinically meaningful in a severely debilitated cohort, many of whom are functioning near their physiologic limits. Importantly, there was no evidence of oxygen desaturation or worse-ning gas exchange during the six-minute walk test, which is reassuring with respect to ventilation–perfusion mis-match, a known limitation of systemic vasodilator therapy.

The dual mechanism of ensifentrine provides a compelling theoretical rationale for its application in COPD-associated PH. PDE3 inhibition increases intracellular cyclic adenosine monophosphate (cAMP), producing smooth muscle relaxation in both the airways and pulmonary vasculature, leading to bronchodilation and mild pulmonary vasodilation without systemic hypotension. PDE4 inhibition exerts anti-inflammatory effects, potentially attenuating cytokine-mediated vascular remodeling and pulmonary arterial stiffness, key drivers of elevated PVR in Group 3 PH [15]. Importantly, ensifentrine’s inhaled formulation delivers the drug directly to the lungs, maximizing local efficacy while minimizing systemic side effects, including hypotension.

Our observations suggest that ensifentrine may improve pulmonary hemodynamics, functional outcomes, and right ventricular performance in patients with severe COPD-PH, although the relative contribution of direct vascular effects *versus* improvements in airflow and inflammation with secondary effects on alveolar recruitment and ventilation-perfusion matching remains uncertain. It is plausible that enhanced bronchodilation and anti-inflammatory effects may have mitigated regional hypoxic pulmonary vasoconstriction, resulting in improved pulmonary vascular resistance.

Notably, this is, to our knowledge, the first report evaluating ensifentrine in patients with COPD-associated PH confirmed by right heart catheterization.

Data from the ENHANCE trials demonstrated improvements in lung function, symptom burden, and quality of life with ensifentrine in patients with moderate-to-severe COPD over 24 weeks [7]. In contrast, our patients did not exhibit significant improvement in FEV_1_. This is likely because they had very severe obstructive physiology with advanced emphysema as the predominant phenotype, in contrast to the trial population. Furthermore, our study was observational and included a very small number of subjects who likely had minimal or no residual reversible airflow limitation.

Combination therapy with ensifentrine and roflumilast also appeared to be well tolerated, without the adverse effects typically associated with PDE4 inhibition, such as weight loss, depression, or hepatotoxicity. One patient not included in the analysis experienced vivid dreams and nightmares, requiring discontinuation of therapy within one week, underscoring the need for individualized monitoring. It remains unknown whether the combination of roflumilast and ensifentrine is beneficial, and further evidence is required to support such an approach. If considered as salvage therapy in end-stage COPD, these agents should be introduced sequentially rather than simultaneously, with close monitoring for neuropsychiatric and gastrointestinal side effects within a shared decision-making framework.

Following six months of ensifentrine therapy, two patients in this cohort did not tolerate sildenafil due to adverse effects, and three reported no clear subjective improvement. However, the absence of formal objective evaluation in these individuals, combined with the severity of their disease, may have limited the detection of meaningful clinical benefit.

Overall, while this study is constrained by its small sample size, retrospective design, and lack of a control group, the findings provide preliminary insight suggesting that ensifentrine may be associated with favorable pulmonary hemodynamic effects. However, as a small observational case series without a comparator group, no statistical analysis or propensity score matching was possible, limiting definitive conclusions. It remains unclear whether these findings represent a true signal or random variation, and prospective controlled studies are required for clarification. Larger randomized trials are warranted to validate these observations and determine whether ensifentrine could be integrated earlier (*e.g.*, in patients with PVR >3 Wood units) into treatment algorithms for this challenging and under-treated population.

## 5 STRENGTHS AND LIMITATIONS

This study has several limitations that warrant consideration. A major limitation is the small sample size (n = 5), which substantially limits statistical power and generalizability. With such a limited cohort, inter-individual variability may strongly influence observed outcomes, and random fluctuations cannot be reliably distinguished from true treatment effects.

In addition, the absence of a control arm precludes differentiation between treatment-related effects and natural disease progression, regression to the mean, placebo effects, or the impact of concurrent interventions. Although patients were maintained on maximal guideline-directed COPD therapy, changes in symptom management, rehabilitation intensity, or patient behavior during the observation period may have contributed to the modest improvements observed.

Taken together, these limitations indicate that the findings should be interpreted as preliminary and hypothesis-generating. Future research should address these constraints through larger, controlled studies. Furthermore, methodological considerations in observational research, such as immortal time bias, highlight the importance of accounting for time-dependent exposures when assessing treatment effects. Incorporating such methodological safeguards may improve the validity of effect estimates and support the development of more robust conclusions [16, 17].

Although all patients in this study were maintained on stable, guideline-directed COPD therapy for at least 3 months before initiation of ensifentrine, and no major therapy changes occurred during the observation period, it is possible that concomitant therapies may still represent a potential source of confounding inherent to retrospective analyses. The retrospective, non-randomized design introduces inherent limitations, including incomplete data capture, unmeasured confounding, and the inability to establish causality.

A further major limitation is the potential for selection bias due to the exclusion of nine of the fourteen initially enrolled patients. Excluded individuals included those who experienced exacerbations or hospitalizations, were unable to undergo repeat right heart catheterization, had drug intolerance, or experienced intercurrent clinical events. As a result, the analyzed cohort likely represents a subgroup that was more clinically stable, better able to tolerate therapy, and potentially more adherent to treatment.

This selective inclusion may overestimate both treatment effects and safety outcomes, as it does not capture the heterogeneity and clinical complexity of real-world COPD populations, particularly those with frequent exacerbations or multiple comorbidities. The limited representativeness of the cohort, therefore, constrains the generalizability of the findings and suggests that the observed improvements may not reflect typical clinical outcomes. Future studies should prioritize broader and more inclusive recruitment strategies to better define the effectiveness and safety of these interventions in routine practice.

A limitation of this study is that inflammatory biomarkers such as C-reactive protein (CRP), fibrinogen, or sputum inflammatory mediators were not measured. As pulmonary hypertension in COPD may have an inflammatory component, the absence of these data limits the ability to evaluate potential non-hemodynamic (anti-inflammatory) effects of ensifentrine. There is a trend toward improvement in some parameters, such as the CAT score and mPAP; however, larger prospective trials are required to determine statistical significance and confirm these findings.

Conducting the study at a single center further limits external validity, as patient characteristics, management strategies, and institutional practices may not reflect those of broader or more diverse populations. In addition, the absence of an untreated or placebo control group precludes definitive attribution of observed improvements to ensifentrine, as these may be influenced by concurrent therapies, natural disease variability, or observer bias. The open-label nature of treatment may also have introduced reporting and observer bias, particularly for subjective outcomes such as dyspnea scores and patient-reported symptoms.

While right heart catheterization remains the gold standard for hemodynamic assessment, measurements in patients with severe obstructive lung disease may be affected by intrathoracic pressure variability, potentially reducing precision.

Despite these limitations, this study also has several strengths. All patients had pre-capillary pulmonary hypertension confirmed by right heart catheterization, ensuring diagnostic accuracy. The cohort was well characterized clinically and phenotypically, with detailed functional, imaging, and hemodynamic assessments. Patients were maintained on maximized guideline-directed COPD therapy, allowing the effects of ensifentrine to be assessed in the context of optimized standard care. Finally, systematic collection of safety outcomes, including tolerability of combined ensifentrine and roflumilast therapy, provides practical insights for real-world clinical use.

The observed improvements in six-minute walk distance and patient-reported outcomes may have broader implications for overall well-being. Although this study was not designed to assess mental health outcomes, improved functional capacity may indirectly influence psychological well-being. However, these associations remain speculative, and future studies should evaluate mental health outcomes directly in this population.

Taken together, this study is hypothesis-generating in a population with very limited therapeutic options and underscores the need for larger, prospective, multi-center randomized controlled trials to validate these findings.

## 6 FUTURE DIRECTIONS

The findings from this retrospective analysis highlight several important directions for future research. Foremost, larger multi-center randomized controlled trials are needed to rigorously evaluate the role of ensifentrine in patients with COPD-associated pulmonary hypertension, ideally employing co-primary endpoints that include both hemodynamic response and functional capacity. Beyond clinical outcomes, mechanistic studies using advanced imaging modalities such as cardiac magnetic resonance imaging, perfusion imaging, and gas exchange assessments would provide valuable insights into the drug’s potential effects on pulmonary vascular remodeling and right ventricular function.

Additionally, future investigations should focus on identifying patient subgroups most likely to benefit from PDE3/4 inhibition. Stratification by emphysema-dominant *versus* chronic bronchitic phenotypes, as well as prior responsiveness to phosphodiesterase inhibitors, may help identify clinically meaningful patterns of treatment response. Incorporating biomarker-driven stratification approaches may further refine patient selection and optimize therapeutic benefit. Together, these avenues of research will be critical for defining the role of ensifentrine in the management of this complex and under-treated population.

## CONCLUSION

In this retrospective study, nebulized ensifentrine was safe and was associated with modest improvements in pulmonary hemodynamics, functional capacity, and dyspnea in patients with severe COPD and pre-capillary PH. These findings highlight the potential of ensifentrine as a dual-modality therapy targeting both airway obstruction and pulmonary vascular dysfunction. However, due to the small sample size and absence of a control group, the certainty of these findings is limited. Larger prospective studies are needed to confirm these preliminary observations.

## Figures and Tables

**Fig. (1):**
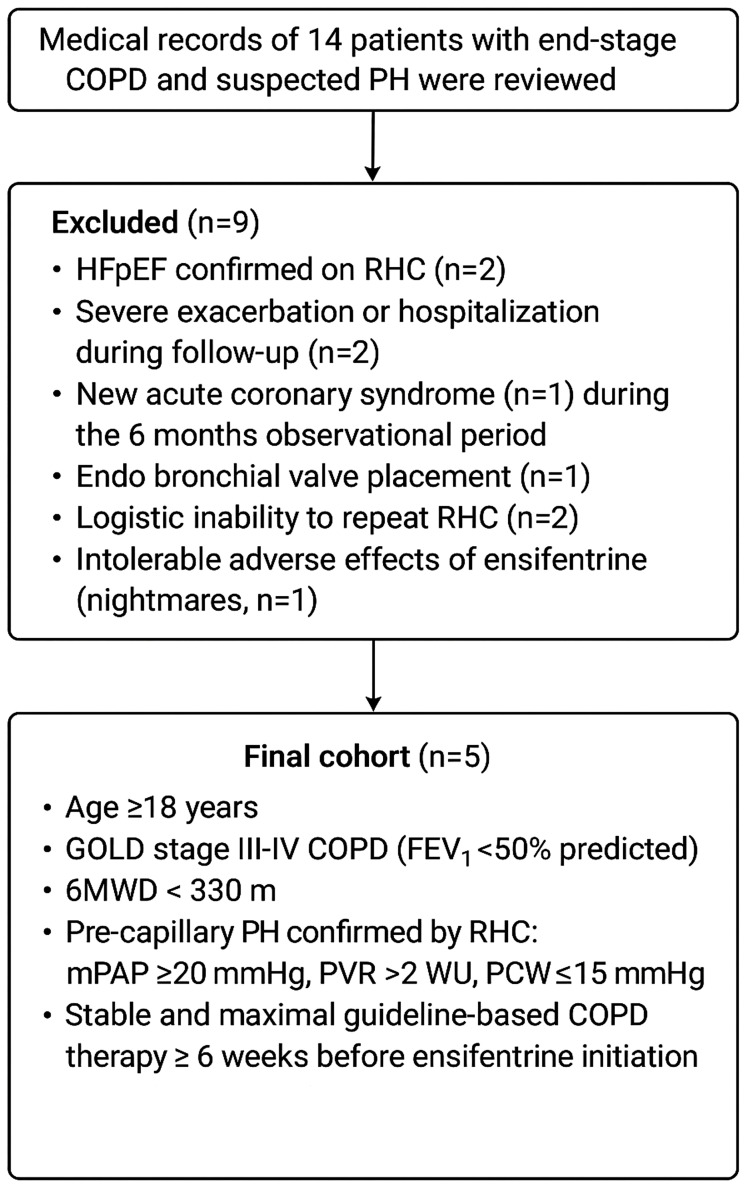
Study flow chart of the patients included in this study.

**Fig. (2):**
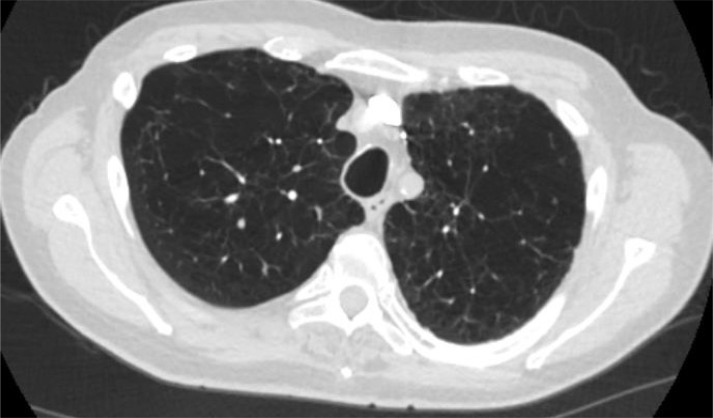
Axial chest computed tomography (lung window) at the level of the trachea in one patient, demonstrating severe emphysema.

**Table 1:** Baseline characteristics of the five patients. All these patients had severe emphysema on imaging and a mean FEV1 of 33%, on maximum medical therapy. See text for further details.

**Baseline Characteristics**	**Mean value (if applicable)**
Age (in years)	76.2
Sex= Male (n/5)	n=3
BMI	19.6
Smoking Hx (in pack years)	47
Charlson Comorbidity Index	9.6
Bronchiectasis (n/5)	0/5
Bronchitis (n/5)	3/5
Emphysema as determined by CT Chest (n/5)	5/5
Exacerbation Hx: moderate (n/5)	2/5
Exacerbation Hx: severe (n/5)	2/5
GOLD Stage E (n/5)	5/5
Hb (g/dl)	14.66
Eosinophil count (cells/uL)	210
FEV1/FVC	33.6
LAMA+LABA (n/5)	1/5
LAMA+LABA+ICS (n/5)	4/5
On Long-term Oxygen therapy (n/5)	5/5
Non-invasive ventilator Rx (n/5)	4/5
Roflumilast Rx (n/5)	4/5
Chronic Azithromycin (n/5)	4/5
Chronic low-dose prednisone (n/5)	3/5
Post Pulmonary rehab (n/5)	4/5

**Table 2:** Functional and hemodynamic outcomes at baseline and 6 months after ensifentrine nebulization.

**Parameter (Mean Value ± SD)**	**Before Ensifentrine**	**6 Months After Ensifentrine**
MMRC Score	3.6 ± 0.54	3.2 ± 1.09
CAT score	23 ± 8	21.6 ± 8.2
6MWT in meters	224 ± 16.7	255.4 ± 25.7
PFT: FEV1 (% predicted)	33.4 ± 3.78	33.2 ± 3.49
PFT: FVC (% predicted)	80 ± 7.81	80.2 ± 7.46
PFT: DLCO (% predicted)	29 ± 4.06	31.2 ± 4.65
Echo: ePASP (mm of Hg)	62.4 ± 8.5	53.8 ± 5.63
Echo: TAPSE (cm)	1.42 ± 0.16	N/A
RHC: mPAP (mm of Hg)	53.2 ± 7.88	51.6 ± 6.06
RHC: PCWP (mm HG)	10.2 ± 1.48	9.8 ± 1.30
RHC: PVR (wu)	9.5 ± 1.94	8.5 ± 2.24
RHC: CI (L/min/m2)	2.5 ± 0.56	2.62 ± 0.44

## Data Availability

The data supporting the findings of the article is available in the Zenodo Repository at10.5281/zenodo. 20844682.

## LIST OF ABBREVIATIONS

PH: Pulmonary Hypertension
COPD: Chronic Obstructive Pulmonary Disease
RHC: Right Heart Catheterization
PVR: Pulmonary Vascular Resistance
TAPSE: Tricuspid Annular Plane Systolic Excursion
